# Polymorphic Conversion of Furosemide after Crystallization in Aqueous Polymeric Solution: Crystallite Analysis Using Scherrer vs. Williamson-Hall Equation and Drug Dissolution

**DOI:** 10.34172/apb.025.45868

**Published:** 2025-09-04

**Authors:** Mouli Das, Sk Habibullah, Alisha Khandelwal, Rakesh Swain, Tanisha Das, Subrata Mallick

**Affiliations:** ^1^Department of Pharmaceutics, School of Pharmaceutical Sciences, Siksha ‘O’ Anusandhan, India; ^2^Department of Pharmaceutics, Utkal University, India

**Keywords:** Furosemide, Polymorphic conversion, Scherrer equation, Williamson Hall equation, Drug dissolution

## Abstract

**Purpose:**

Furosemide (FUR) is a potent loop diuretic, practically water-insoluble drug and also known to exhibit polymorphic form I (most stable), form II (metastable), and form III (metastable), and also a poorly stable amorphous form. FUR was crystallized using aqueous polymeric solutions and the effect of crystallite properties on polymorphic transformation and dissolution was studied.

**Methods:**

FUR crystal was prepared using aqueous solution of HPMC, CMC, MC, or, PVA as non-solvent (as FH, FC, FM, and FP respectively), and spectral analysis and in vitro dissolution was performed.

**Results:**

FTIR study of FH and FM exhibited only two peaks (3342.99 and 3250.43 cm-1), considering form II, whereas, FP and FC presented three distinct peaks between 3400-3200 cm-1 (3397.95, 3348.78, and 3280.32 cm-1) corresponding to asymmetric sulfonamide-NH, secondary amine-NH, and symmetric sulfonamide-NH stretching, confirming the presence of form-I. Further, XRD and DSC also confirmed the polymorphic identity of the crystal forms. Crystallite size analysis using the Scherrer equation and Williamson-Hall plot revealed a significant reduction in the size of all crystal products compared to pure FUR, with an associated increase in dislocation density, suggesting enhanced structural imperfections. All prepared crystals demonstrated markedly improved dissolution profile relative to the pure drug. Furthermore, stability studies under accelerated condition (40 °C/75% RH, 3 mo) confirmed the retention of respective polymorphic forms without any noticeable changes (FH and FM stayed in form II and FC and FP stayed in form I).

**Conclusion:**

This study concluded that the stable polymorphic form of furosemide crystal was produced using aqueous polymeric solution with improved dissolution.

## Introduction

 Furosemide (FUR), a potent loop diuretic, is used to treat hypertension as well as edematous disorders associated with cardiac, renal, and hepatic failure.^1^ FUR is practically water-insoluble^2,3^ exhibiting poor permeability^4^ and also low oral bioavailability (50 %).^5^ Moreover, FUR is known to exhibit three polymorphic forms as form I (most stable and commercially used), form II (metastable), form III (metastable), and an amorphous form of lower stability.^6^ Polymorphic form differs in molecular packing and/or arrangement within the framework of the crystal.^7,8^ The physicochemical properties of the polymorph lead to variations in solubility, bioavailability, and stability.^9,10^ Polymorphic transformation of FUR during solid state milling has been observed by Wang et al. 2023. Their study revealed that form I and III transitioned directly into the amorphous state, whereas form II initially transformed into form I before progressively losing its crystalline structure and converted to an amorphous form.^11^ Phase transition studies also indicated that the metastable form-II changes into form-I during both grinding and slurry experimentation.^3^ In general, selecting an appropriate solid-state form of the drug, such as polymorph, hydrate, solvate, salt, or cocrystal, can significantly influence its physicochemical and pharmacokinetic characteristics, including its compressibility, stability, dissolution behaviour, bioavailability.^12,13^ Polymorphism has also been observed in FUR solvates (with DMSO and DMF) after varying crystallization conditions.^14^

 Crystal design and engineering play a vital role in influencing drug dissolution, stability, bioavailability, and formulation processability. Nucleation and crystal growth critically influence the physicochemical and structural characteristics during solution-state crystallization.^15^ Altering crystallization solvent significantly modified crystal habit or external shape, and also influenced bulk properties, as demonstrated in case studies involving ibuprofen.^16^ Another key area of research involves the role of additives or impurities in modulating crystallization by either promoting or hindering growth.^17-20^

 Various hydrophilic polymers can influence crystal habit, stabilize amorphous and metastable phases of the specific polymorphic form.^15^ These hydrophilic polymers may absorb on the drug crystal surface offering more sites for interaction with the polymers leading to improved or declined crystal growth.^21^

 Polymorphic transformation of the drug could be possible using hydrophilic polymeric solution as non-solvent during crystallization by modulating nucleation kinetics, crystal growth, and molecular interactions, particularly hydrogen bonding and steric effects facilitating selectively stabilized distinct polymorphic forms. HPMC, MC, CMC, and PVA as the hydrophilic polymer influenced FUR for polymorphic transformation when used as non-solvent in a recent report from our laboratory.^22^ Chlorpropamide metastable form II has been transformed to form III and again transitioned to stable form A using 2-hydroxybutyl-β-cyclodextrin as reported by Ishiguro et al.^23^ In another study recrystallization of flufenamic acid in the presence of HPMC produced metastable form IV rather than stable form I.^24^ Formation of higher percentage of stable polymorph γ-indomethacin by accelerated heterogeneous nucleation in the presence of PVA is also known from a previous publication.^25^ Cellulose ethers such as methylcellulose (MC) have been shown to modulate polymorphism; co-melting with paracetamol induced metastable form II via inhibition of stable form I crystallization.^26^ Atrovastatin was crystallized using water as a non-solvent after co-dissolving with HPMC in a solvent wherein, a distinct improvement in drug release was observed.^27^ PVP-induced celecoxib crystals using water as a non-solvent also showed distinctively enhanced drug release.^28^ Hydrophilic polymer has in general the ability to interfere with the nucleation and crystal growth particularly in composite crystal.^29^

 To customize the problem associated with the FUR several methods has been used, like solid dispersion,^30^ co-grinding with polymeric materials,^31^ complexation with cyclodextrins,^32^ self-emulsifying drug delivery systems,^33^ co-crystallization,^34,35^ and nanoparticles.^36^ But crystallization of FUR in aqueous polymeric solution has rarely been explored for transforming polymorphic form I (stable) to stable polymorphic form II with improved dissolution. The present work could be supported by classical nucleation theory (CNT) based polymer-induced crystallization forming primarily a new thermodynamic phase or a metastable form. Moreover, competitive nucleation to multiple polymorphs in supersaturated solutions may be influenced by polymer concentration and interaction dynamics.^37^ HPMC, CMC, MC, and PVA might have increased the interfacial free energy barrier modifying the nucleation kinetics and stabilizing metastable polymorphs through steric hindrance and hydrogen bonding interactions with drug molecules.

 FUR was crystallized using aqueous polymeric solutions (HPMC, MC, CMC, and PVA) as non-solvent. Stable polymorphic transformation was observed with HPMC and MC (form I to form II) on the other hand all crystallized products improved dissolution. This novel technique of producing FUR form II could be commercially relevant with improved bioavailability potential.

## Materials and Methods

###  Materials

 We received a complimentary sample of FUR (form-I) from Zydus Cadila, Ahmedabad, India. HPMC and MC were procured from Burgoyne and Co. (Mumbai, India). PVA and CMC were obtained from HIMEDIA Laboratories Pvt. Ltd., Mumbai, India.

###  Method of Preparation

 FUR was crystallized in the presence of 0.5 % w/w aqueous polymeric solution (PVA, CMC, MC, and HPMC) as a non-solvent. In a vessel, 1000 mg of FUR was dissolved in 20 mL of ethanol. The prepared polymeric solution (about 300 mL) was added slowly with a thin stream to the aforementioned ethanolic drug solution placed in an ice bath. The vessel containing drug crystal was then placed in the refrigerator, and cooling was continued for about 24 h. Prepared crystals were then filtered using Whatman® filter paper (Grade 40 circle) and dried overnight at 40 °C in a hot air oven and preserved in an airtight bottle till further analysis.

###  Scanning electron microscope (SEM) 

 A scanning electron microscope (SEM; Zeiss, Germany) was used to analyze the surface morphology of the crystal products. Before imaging, the samples were affixed to aluminium stubs using double-sided carbon adhesive tape. A thin gold coating was then applied under vacuum using a sputter coater to improve conductivity and avoid charging effects during electron beam exposure. Surface features were visualized in detail by capturing micrographs at multiple magnifications and appropriate resolution.

###  FTIR spectroscopy

 The polymers and the powder samples were analyzed using Jasco-FTIR-4600, Japan. The samples were analyzed in the range of 4000-600 cm^-1^. The equipment was used and operated in the principle of ATR mode. An average of 32 scan /2 cm^-1^ was reported.

###  Thermal analysis 

 The thermal properties of the crystallized products were inspected using a differential scanning calorimeter (TA Instruments, USA) at a thermal scan of 10 °C/min from 30 to 250 °C. To carry out the experiment small amount of FUR crystal was sealed in aluminum pan, with maintaining the nitrogen flow (50 mL/min). TA Universal Analysis 200 software was used for all the formulations.

###  X-ray powder diffraction (XRD)

 Diffraction pattern of pure FUR and the crystallized products were analyzed using XRD (Model: Rigaku, Ultima IV, Japan). For the X-ray source, the D/teX Ultra 250 1D detector has been used with a voltage of 30 kV and 40 mA current. The scanning was done in the 2Ɵ range of 5–70° with a scanning speed of 20° per minute.

###  In vitro drug dissolution 

*In vitro* drug dissolution was carried out using paddle type dissolution apparatus (Dissolution Tester, USP; Model: TDT06L, Electrolab, India).^38^ After passing through 44-mesh sieve, 10 mg of the crystal product was accurately weighed and placed into the vessel with rotating paddle (50 rpm) containing 900 mL dissolution fluid (pH 1.2, 6.8) and also in distilled water (pH 6.4, non-buffered) for 3 h maintained at 37 ± 1 °C. At a predetermined time interval, 10 mL sample was withdrawn and filtered through a 0.45 μm syringe filter. Drug content was estimated spectrophotometrically (Model: Shimadzu, 1900i) at λ_max_ 229 nm. The study was done in a triplicate manner (n = 3).

###  Stability testing of the product

 The pure drug sample as well as the crystal products were stored in a controlled condition of 40 °C at 75 % relative humidity. The samples were removed after 3 month and analyzed to examine the possible changes in crystal form.

###  Statistical analysis

 Results are expressed as the mean ± standard deviation based on experiments conducted in a triplicate.

 The statistical method commonly used for dissolution profile comparison is the “similarity factor” (*f*2) and “difference factor” (*f*1) analysis. The *f*1 and *f*2 values calculated by following equation:


(1) $$f1=\{[\overset{n}{\underset{t=1}{\sum }}|R_{t}-T_{t}|]/[\overset{n}{\underset{t=1}{\sum }}R_{t}\}\times 100$$

And,


(2) $$f2=50.\log\left\{{\left[1+\frac{1}{n}\sum_{t=1}^{n}{\left(R_{t}-T_{t}\right)}^{2}\right]}^{-0.5}\times 100\right\}$$

Where, R_t_ is the percentage drug dissolved for the refence batch at time t, T_t_ is the percentage dissolved drug for the test batch and n is the number of time points.

 Statistical evaluations for crystallite size were carried out using GraphPad Prism software (version 8.0.2). One way ANOVA followed by Tukey’s post hoc test, was applied to assess significance.

## Results and Discussion

###  SEM

**Figure 1:**
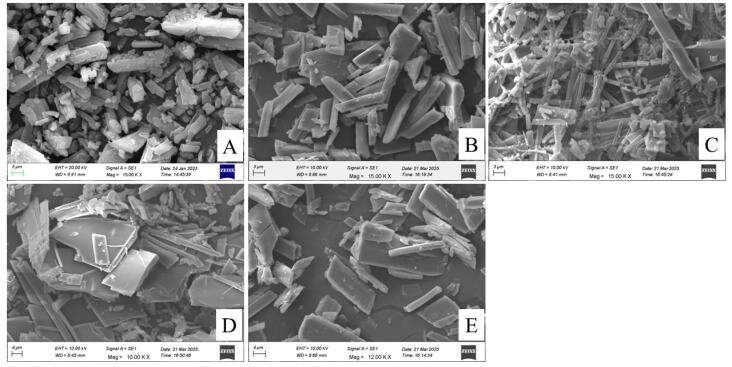
Scanning Electron Microscopy of (A) pure FUR; (B) FC; (C) FP; (D) FM; (E) FH

 SEM analysis revealed that the crystal morphology of pure FUR and formulated crystals FC and FP (Figure 1) is similar to the hexagonal tube-shaped crystal as reported by Garnero et al.^39^ Both the crystal geometry and size of other samples (FH and FM) are significantly different from the pure drug (FUR).

###  FTIR analysis

**Figure 2:**
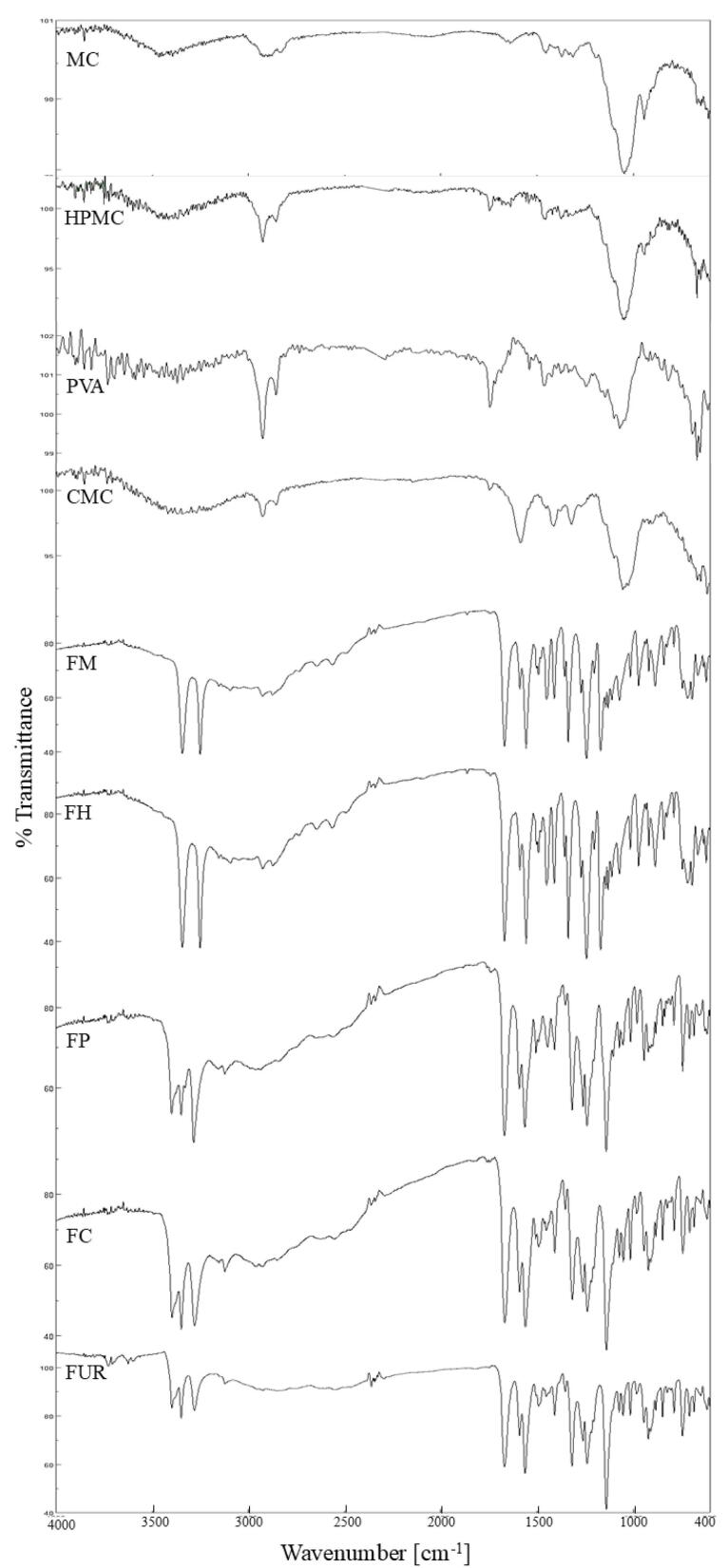
FTIR spectra of pure drug (FUR) and formulated crystals (FM, FH, FP, and FC) along with polymer-only control (MC, HPMC, PVA, and CMC)

 The FTIR analysis was performed to assess the interaction of the pure drug with the recrystallized products. FTIR spectra of the recrystallized product are shown in Figure 2. Polymer-only controls for each polymer used (HPMC, CMC, MC, and PVA) showed their usual characteristic peaks due to their chemical bonds and functional groups in the FTIR spectra (Figure 2). No significant peak shift, and new peaks were found. That means the drug and polymers did not chemically react with each other. The chemical structure of FUR contains sulfur groups. It was quite expected that sulfonamide stretching bands would be found in the region of 1400-1000 cm^-1^ wavenumber.^40^ Sharp, distinctive peaks at 3396.03, 3348.78, 3220.32, 1667.16, 1599.17, 1316.18, 1260.25, 1238.08, 1138.76, and 742.46 cm^-1^ were visible in the spectrum of pure FUR. The characteristic peak at 1138.76 cm^-1^ is due to the stretching vibration of the symmetric SO_2_.^40,41^ In addition, other signals are observed at 3396 (asymmetric N-H), 3349 (symmetric N-H), 1666 (C = O), 1599 (C = C), 1316 (asymmetric SO_2_), 1260 (carboxylic acid, C-O), 1238 (Furan ring C-O-C), and 742 cm^−1^ (C-Cl)^40,42-44^ The peak at 3220 cm^-1^ may be due to the stretching vibration of SO_2_NH_2_.^45^ From this analysis, it was observed that recrystallization with polymers like CMC and PVA does not show polymorphism. However, polymers like HPMC and MC change the polymeric form of the FUR. Notable spectral variations were observed in the FM and FH samples compared to the pure drug, indicating structural polymorphic transitions. FUR was exhibited in more than one polymorph form, in which Form I was stable, and pure drugs come under this Form.^46^ The presence of 3 resonance peaks at positions 3397.95, 3348.78, and 3280.32 confirms the form I.^3^ Three sharp resonance peaks at 3397.95, 3348.78, due to NH and 3280.32 were due to NH2.^41^ A similar observation was found in the case of FP and FC. In the region of 3400 to 3200 cm^-1^ three distinguishable peak was found, which confirms FP and FC exhibited in the form I. In FM and FH regions (3400 to 3200 cm^-1^) there were 2 distinguished peaks found at wavelength region 3342.99 (secondary amine NH) and 3250.43 (symmetric sulfonamide NH), respectively. Peak at this position confirms the FUR form II. Hence from the findings, it was confirmed that during the crystal formation, FP and FC exhibited in the pure drug polymorphs forms I and FH and FM are found in polymeric form II.

###  XRD analysis

**Figure 3:**
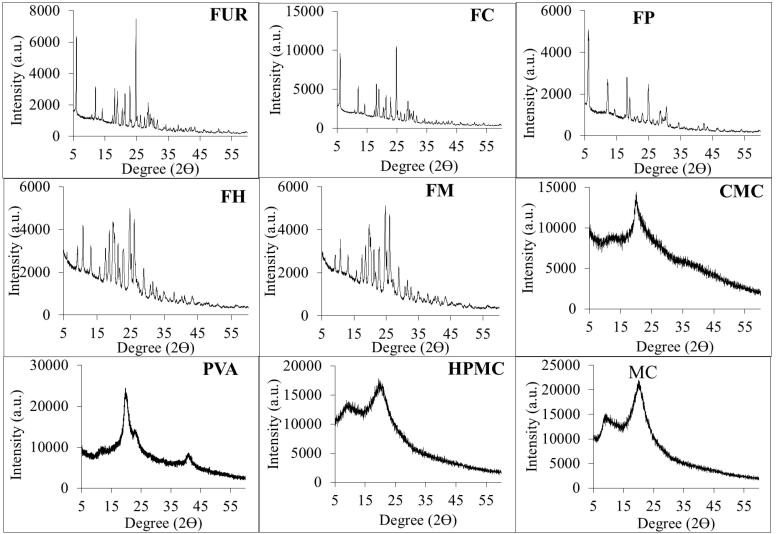
X-Ray Diffractogram of pure FUR and prepared crystals (FC, FP, FH, and FM) along with polymer-only control (CMC, PVA, HPMC, and MC)

 To know the structural analysis and evaluate the crystalline size XRD analysis has been done. Diffraction peak changes in size or shape point to crystal lattice flaws. From this study, the FUR shows 2θ values of 6.01, 10.5, 14.82, 24.8, 21.3, and 22.86 (Figure 3). Which supports the FUR was present in the form-I.^6^ XRD patterns of individual polymers displayed broad, diffuse halos without any sharp, well-defined Bragg peaks, which is indicative of their predominantly amorphous nature.^47^ The same type of crystal packing was observed in the FM and FC, which confirms that FP and FC are present in the form-I. However, differences in the relative intensities of specific peaks between FUR and the FUR crystal products treated with polymers were found in FM and FH. This may be explained by not only a change in crystallographic plane orientation caused by the interference of polymers but also a change in the polymeric form.^6,48^ With the FUR peak, the recrystallized crystal product (FH and FM) shows an intense peak at positions 24.6, 26.1, and 19.8, which confirms the form-II polymeric form.^6^ The peak intensities for the FUR crystal products were found to have altered dramatically, despite the fact that the peak positions had not changed much in FC and FP. In the polymer-treated FUR crystal products, there was a noticeable widening of several peaks, which points to imperfections in the unit cell’s molecular arrangements.

 To clarify the crystal parameters of the experimental FUR crystal products, such as crystal size, strain, and dislocation density, Debye-Scherrer’s formula was adopted.

 For the calculation of crystallite size, the below equation is used.


(3) $$D=\frac{K\;\lambda }{\beta \cos\theta }$$

Where *D* represents crystallite size, *K* value was 0.9, λ of X-ray was found to be 0.1541, and *β* represents the FWHM in radian.

 To evaluate the strain in lattice below equation is used


(4) ε=β/tanθ

Dislocation density (*δ*), which indicates the degree of flaws or faults in the crystal sample, may be calculated by using the following formula


(5) $$\delta =1/D^{2}$$

The reduction in crystallite size (*P* < 0.05) suggests the formation of less ordered domains, possibly due to surface interactions with the polymer matrix. This increases surface area and enhances dissolution.^49^

 The traditional Scherrer method does not account for instrumental and strain contributions to XRD peak broadening. In a diffractogram, peak broadening generally arises from three primary factors: crystallite size broadening, instrumental broadening, and lattice strain broadening. Since these factors contribute collectively to the overall broadening of diffraction peaks, it is essential to minimize the instrumental broadening for accurate analysis.

 In the Williamson-Hall (W-H) approach, peak broadening is separated into contributions from crystallite size, as lattice strain, as illustrated in equation


(6) $$\beta_{sample}=\beta_{crystallite\;size}+\beta_{lattice\;strain}$$

The uniform deformation model (UDM) is the most widely applied version of the W-H method. In this model, the contributions from crystallite size and lattice strain are determined using following equation:


(7) $$\;\beta_{crystallite\;size}=\frac{K\lambda }{Dcos\theta }$$

(8) $$\beta_{lattice\;strain}=4\varepsilon tan\theta =4\varepsilon \frac{sin\theta }{cos\theta }$$

Where, *ε* denotes lattice strain

 The W-H equation is expressed as by combining equation 7 and 8:


(9) $$\beta \;cos\theta =\frac{K\lambda }{D}+\varepsilon .4\;sin\theta$$

**Figure 4:**
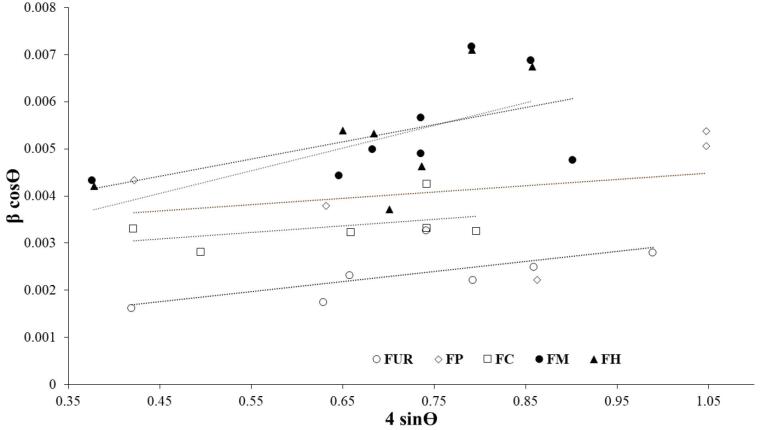
Strain and crystallite size determination by Williamson-Hall plot

A W-H plot is constructed by plotting 4 *sinθ* on the *x*-axis and *β cosθ* on the *y*-axis (Figure 4) for the prepared crystals in the presence of various polymers. From the linear fit, the *y*-intercept was used to determine the crystallite size (*D*), while slope provides the microstrain (*ε*) in the crystal lattice (Table 1).

**Table 1:** Properties of furosemide crystallites developed in aqueous polymeric solution

**Crystal code**	**Polymer solution (0.5 %)**	**FWHM**	**Scherrer**			**Williamson-Hall**	
			**Crystallite size (D) (nm)**	**Strain×10**^-4^** (ε)**	**Dislocation Density (nm**^-2^**)**	**Crystallite size (D) (nm)**	**Strain×10**^-4^** (ε)**
FUR	None	0.131 ± 0.034	74.29 ± 11.93	40 ± 10	0.0008 ± 0.0002	173.37	21
FH	HPMC	0.276 ± 0.100	29.21 ± 6.19	70 ± 20	0.0051 ± 0.0022	72.99	48
FC	CMC	0.180 ± 0.016	43.87 ± 5.95	60 ± 40	0.0021 ± 0.0005	55.48	14
FM	MC	0.314 ± 0.064	30.38 ± 9.05	80 ± 10	0.0052 ± 0.0032	49.53	36
FP	PVA	0.273 ± 0.047	24.38 ± 4.97	120 ± 90	0.0073 ± 0.0032	44.74	13

 The concurrent increase in microstrain and calculated dislocation density suggested that the presence of polymer (HPMC, CMC, PVA, and MC) brought about lattice distortions and defect structures during the process of crystallization. These structural imperfections are known to elevate the internal energy of the crystal lattice, thereby reducing lattice stability and facilitating enhanced wettability and solvent penetration contributing improved dissolution behaviour.^50^

###  DSC analysis 

**Figure 5:**
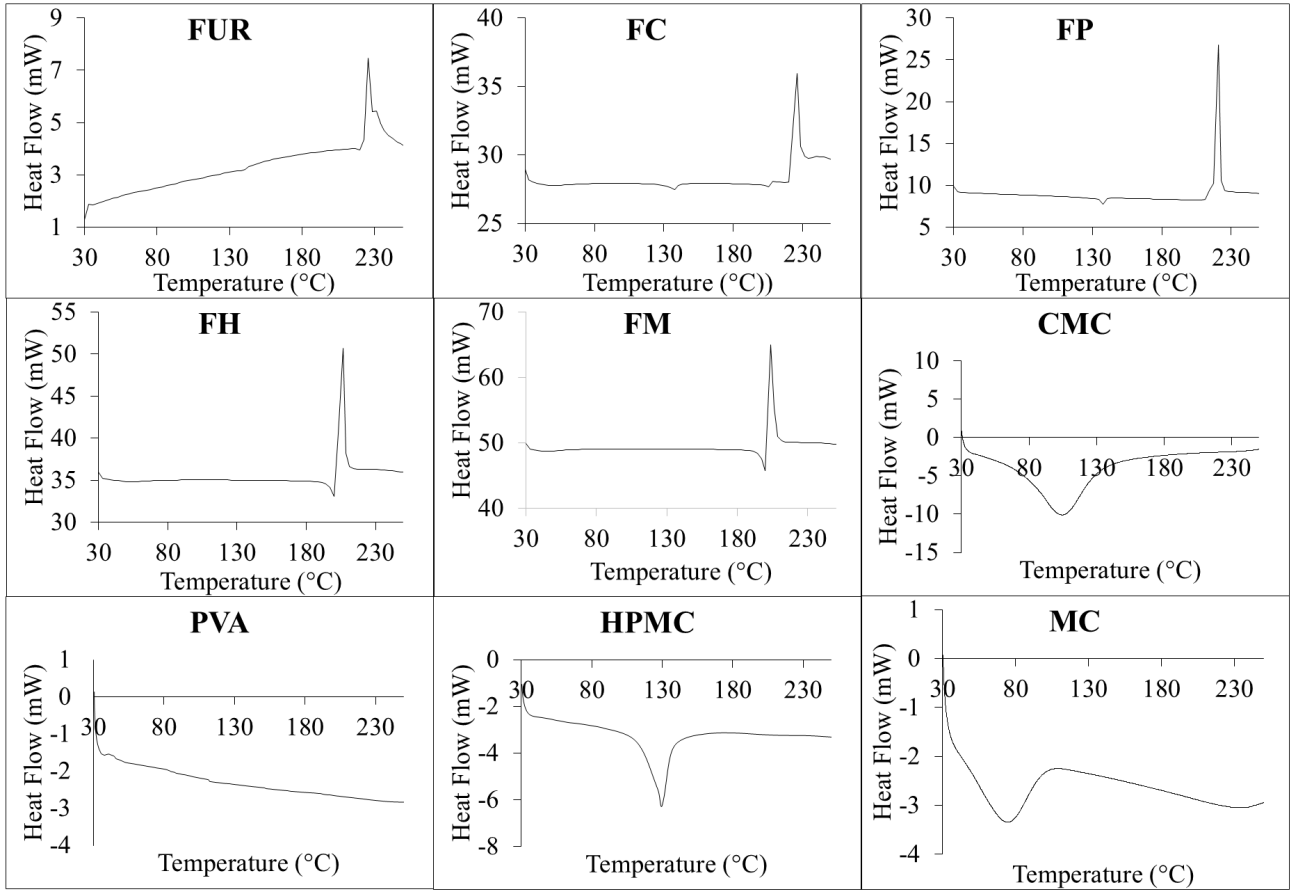
DSC thermogram of drug alone (FUR) and prepared crystals (FC, FP, FH, and FM) along with polymer-only control (CMC, PVA, HPMC, and MC)

 FUR showed a distinctive, strong exothermic peak at 226.35 °C, which not only connected to the melting point of the drug but also showed the crystallinity form of the drug.^43^ FUR-treated polymers show exothermic peaks and migrate toward a low temperature. For FH, FP, FC, FM, and pure drug, the melting exothermic degradation peak was in order 205.55, 221.01, 225.01, 203.26, and 226.35 °C. DSC thermogram of the polymers alone exhibited a broad endothermic peak below 150 °C, as displayed in Figure 5. This peak is typically attributed to the loss of absorbed moisture or the glass transition of the polymer matrix.^51-53^ These transitions are not related to any melting or decomposition events. And these events are well below the melting point of FUR. The clear separation of thermal events confirms that there is no thermal overlap or interference between the polymer and the drug. Additionally, no new peaks were observed, indicating the absence of any strong chemical interactions or incompatibility between the drug and the polymers.

 Pure drug, along with crystal products FP and FC, shows the same pattern that the drug exhibits in Form-I. In FH and FM, the crystal first undergoes a fusion process, which suggests that FH and FM are present in Form-II.

 The melting enthalpy of pure FUR was recorded as 28.43 J/g. in comparison, the enthalpy values of recrystallized formulations observed as: FH: 76.29 j/g, FC: 28.20 J/g, FM: 80.14 J/g, and FP:62.98 J/g. These variations again suggested altered crystalline arrangements. The higher ΔH value of FH and FM might have the indication of the presence of more ordered crystal structure or enhanced lattice energy due to the influence of specific polymeric solution as non-solvent during crystallization. Polymorphic pureness denotes the extent of stable crystal structure of the anticipated polymorphic form and free from other forms. In that respect present results indicated the purity of formulated crystals compared to pure drug.

###  In vitro dissolution study

**Figure 6:**
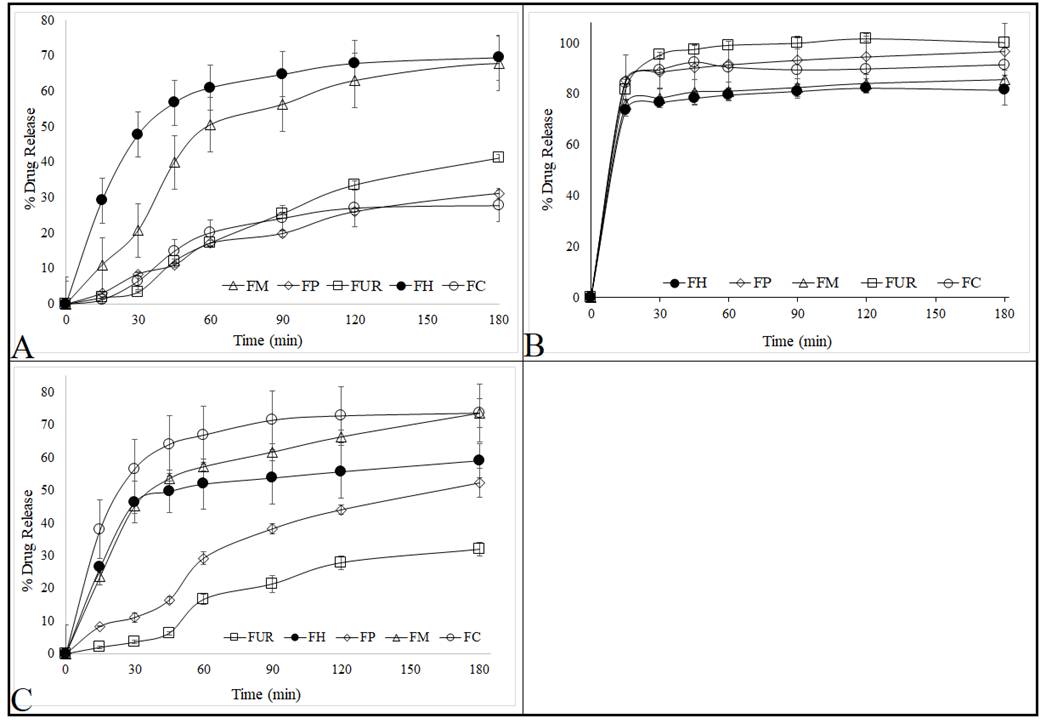
In vitro furosemide dissolution profile (n = 3, means ± SD) of the crystallized product in (A) pH 1.2, (B) pH 6.8, and (C) distilled water (pH 6.4)

Figure 6 illustrates the drug dissolution profile of pure drug and crystal formulations carried out at pH 1.2, 6.8, and distilled water over a 3-hour period.

 The In-vitro release study of FUR was carried out in medium of pH 1.2, 6.8 and distilled water (pH 6.4). Pure drug (FUR) is poorly soluble at pH 1.2 (pKa 3.8)^54^ and FUR ionizes to COO- at pH 6.8 due to its carboxylic acid group. FUR remained mostly in its non-ionized (COOH) form at pH 1.2 and hampered drug release.^55^ As a result, release of pure drug (FUR) was decreased significantly compared to formulated crystals (FM, FP, FH and FC) at pH 1.2 (Figure 6A) and improved drug release (FUR: 99.57 %) was exhibited relative to others at pH 6.8 medium (Figure 6B). Crystallized product in presence of hydrophilic polymer enhanced wetting and hydration, consequently improved drug release significantly rather than FUR in distilled water (Figure 6C). FH and FM (Form II) showed highest release (76.11 %) among the others due to decreased crystallite size and increased lattice strain and dislocation density at pH 1.2.^48^

 A similarity assessment using the difference factor (f1) and similarity factor (f2) was conducted to evaluate the notable improvement in the in-vitro release of FUR compared to the formulated crystals. In a recent meeting a detailed discussion has been reported on the assessment of dissolution profile comparison using *f*2 and *f*1 analysis.^56^ One paper just published from our laboratory described the improvement of dissolution using *f*2 and *f*1 analysis.^22^ Difference in solubility and dissolution has been evaluated using *f*2 factor.^57^ The obtained f1 and f2 values (Table 2) (greater than 15 and less than 50, respectively) indicated a statistically significant variation in the release profile.

**Table 2:** Calculated difference factor (f1) and similarity factor (f2) of all formulations in different pH

**Formulation code**	**Difference factor (f1)**			**Similarity factor (f2)**		
	**pH 1.2**	**pH 6.8**	**pH 6.4**	**pH 1.2**	**pH 6.8**	**pH 6.4**
FH	123.91	189.55	201.71	11.79	2.56	6.19
FC	189.55	16.69	305.76	2.56	48.36	2.83
FM	27.44	123.91	181.59	44.46	11.79	1.94
FP	16.69	27.45	75.55	49.36	44.46	27.50

###  Stability study

**Figure 7:**
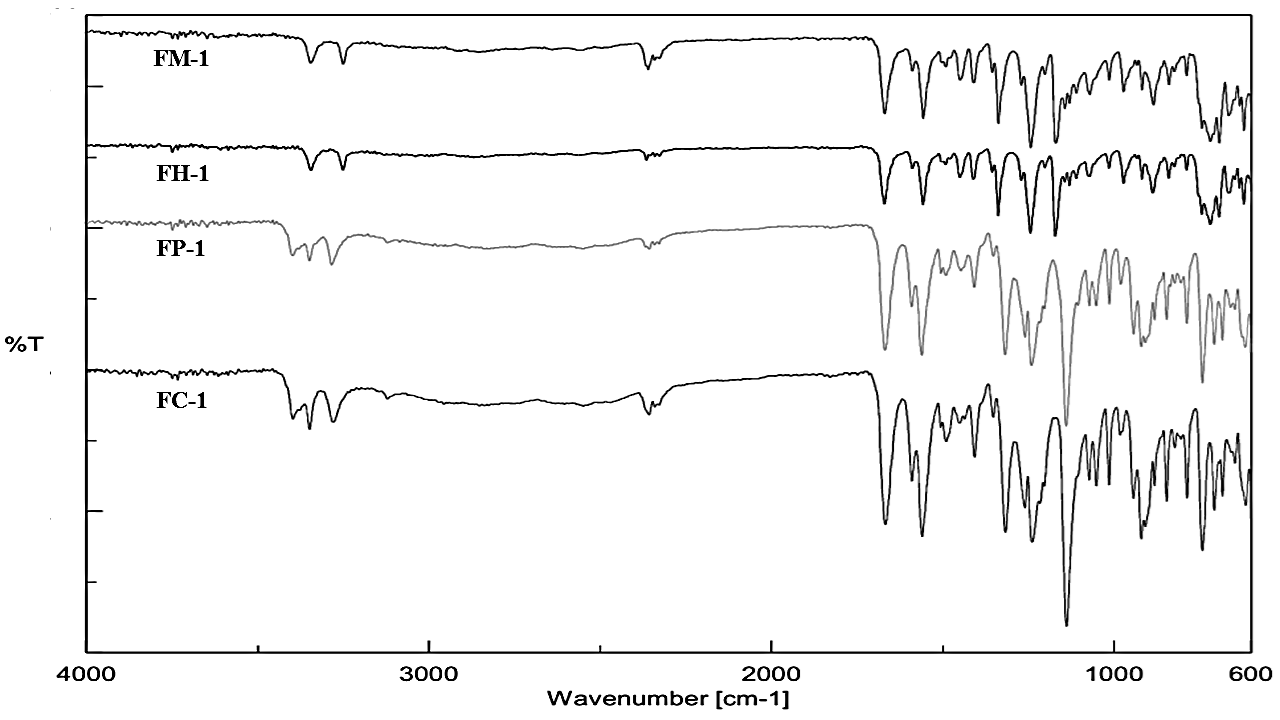
FTIR study of prepared crystals of furosemide after stability study for 90 days (40 °C and 75 % RH)

**Figure 8:**
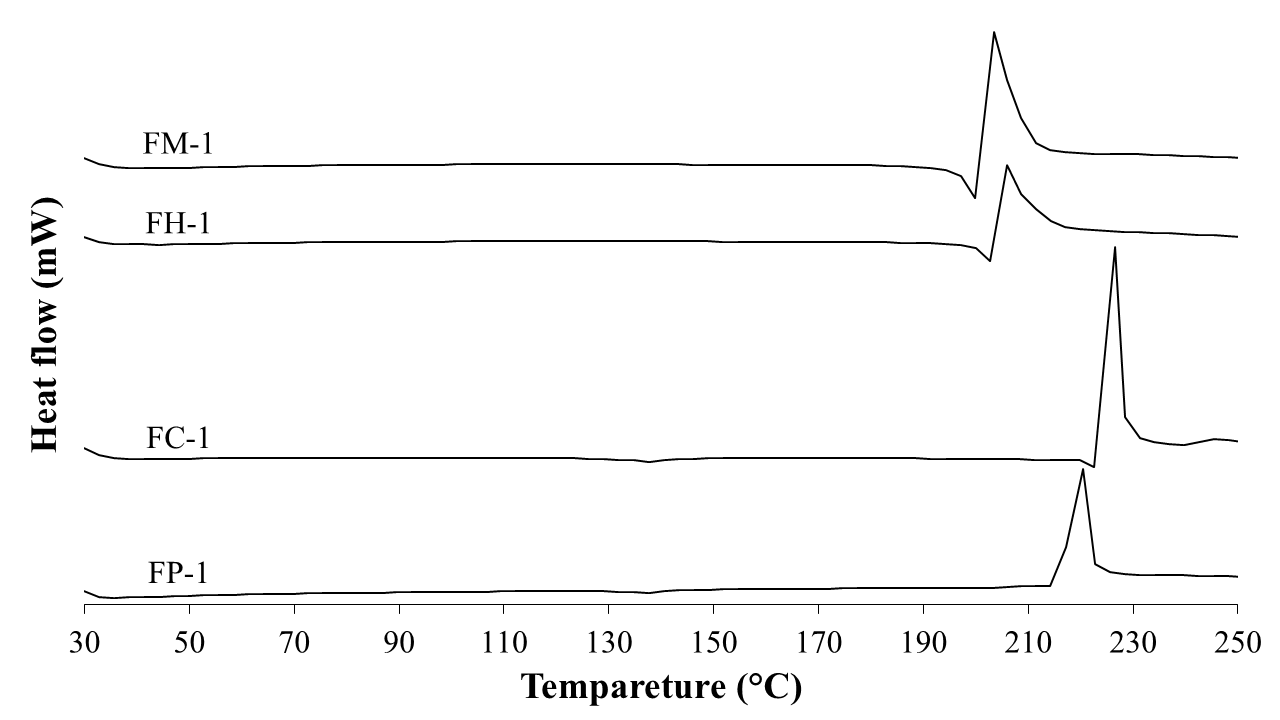
DSC study of prepared crystals of furosemide after stability study for 90 days (40 °C and 75 % RH)

**Figure 9:**
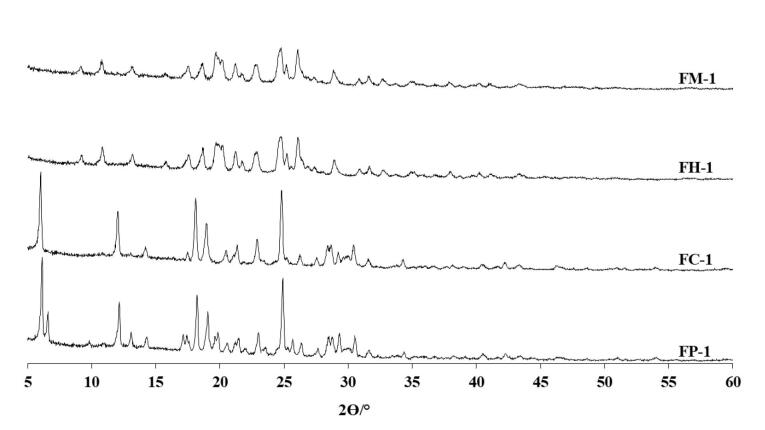
XRD study of prepared crystals of furosemide after stability study for 90 days (40 °C and 75 % RH)

Figures 7, 8, and 9 represent FTIR, DSC, and XRD respectively after the exposure of 40 °C and 75% RH for 3 mo. No significant changes were noticed in FTIR, DSC, and XRD data. Results demonstrated that the form II in crystal product (FH and FM) has not been transformed to any other form. That means, Form II in FH and FM remained stable still after the accelerated storage condition for 3 mo. Whereas, form I in crystal product of FP and FC has also not been transitioned to form II or form III. But, in a recent report phase transformation of the metastable form-II changed into form-I during both grinding and slurry experimentation.^3^

## Conclusion

 In conclusion, FUR was crystallized using aqueous polymeric solution (HPMC, CMC, MC, and PVA) as non-solvent, and stable polymorphic form of crystal product FH (form II), FC (form I), FM (form II), and FP (form I) respectively was successfully prepared. Crystallite size was markedly decreased as understood from both the Scherrer equation and Williamson Hall plot of XRD data compared to pure drug. Dislocation density of the crystal product has been increased due to linear directional imperfection in the crystallite structure. Prominently increased drug dissolution was observed of crystal product rather than pure drug. Accelerated storage condition (3 months) confirmed the stability of all the respective polymorphic form of the crystal product.

## Competing Interests

 All the authors have read the manuscript and nobody has any conflict of interest.

## Data Availability Statement

 Upon the request data will be available.

## Ethical Approval

 Not applicable.
